# Ulcérations buccales et péri-anales: un mode de révélation inhabituel d'une granulomatose avec polyangéite - à propos d'un cas

**DOI:** 10.11604/pamj.2014.18.121.3984

**Published:** 2014-06-06

**Authors:** Neirouz Ghannouchi Jaafoura, Wathek Thaljaoui, Amira Atig, Ahmed Bouker, Mabrouk Khalifa, Fathi Bahri

**Affiliations:** 1 Service de Médecine Interne, CHU Farhat Hached, Sousse, Tunisie

**Keywords:** Granulomatose avec polyangéite, nécrose linguale, ulcère péri-anal, rectorragie, Granulomatosis with polyangiitis, lingual necrosis, perianal ulcer, rectorrhagia

## Abstract

La granulomatose avec polyangéite, est une vascularite systémique rare qui touche avec prédilection les voies aériennes supérieures, les poumons et les reins. L'atteinte cutanéo-muqueuse ainsi que l'atteinte digestive ne sont pas inhabituelles mais elles sont rarement inaugurales de la maladie. Nous rapportons l'observation d'une femme âgée de 57 ans, ayant une granulomatose avec polyangéite multi-systémique avec comme premières manifestations une atteinte cutanéo-muqueuse à type de nécrose de la langue et d'ulcérations péri-anales ainsi que des rectorragies. La présence de signes radiologiques orientant vers une hémmorragie intra-alvéolaire, l'atteinte rénale, l'atteinte neurologique périphérique ainsi que la positivité des C-ANCA de type anti-PR3 ont permis de rattacher les manifestations dermatologiques à cette vascularite. Des manifestations cutanéo-muqueuses atypiques, au cours d'une granulomateuse avec polyangéite, doivent être connues par le clinicien pour un diagnostic et une prise en charge adéquate.

## Introduction

La granulomatose avec polyangéite (GPA) est une vascularite systémique touchant les vaisseaux de petit calibre associant sur le plan anatomopathologique une vascularite nécrosante et une granulomatose [1]. Elle touche principalement les voies aériennes supérieures, les poumons et les reins, rarement le tube digestif [2, 3]. Aussi bien la nécrose de la langue que les ulcérations péri-anales sont des manifestations peu courantes et rarement inaugurales de la maladie. Nous rapportons le cas d'une patiente chez qui des manifestations ano-rectales et buccales ont révélé la maladie.

## Patient et observation

Une femme de 57 ans, sans antécédents pathologiques notables, était hospitalisée en janvier 2013 pour exploration d'un syndrome rectal fait de ténesmes et d’épreintes avec des rectorragies évoluant depuis 20 jours, aux quelles se sont associées une sensation de brulures au niveau de la langue avec apparition d'une ulcération du bord latéral de la langue gênant l'alimentation, saignant au moindre contact et ne s'améliorant pas malgré plusieurs traitements symptomatiques prescrits en ambulatoire. Tous ces symptômes s'associaient à une altération rapidement croissante de l’état général et une faiblesse des membres inférieurs avec paresthésies rendant la patiente grabataire.

**Figure 1:**
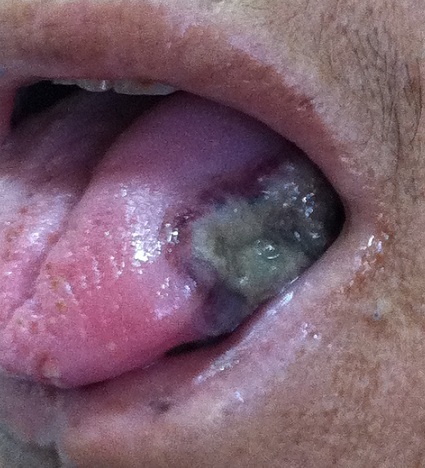
Ulcération nécrotique du bord latéral de la langue

**Figure 2:**
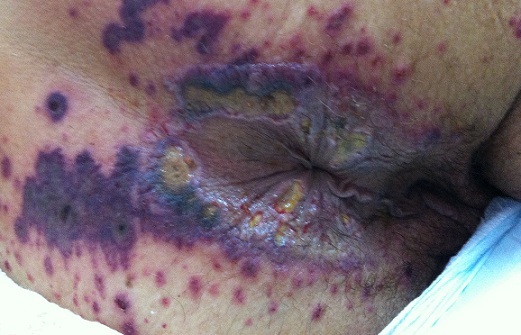
Ulcérations péri-anales avec des lésions purpuriques infiltrées et nécrotiques

A l'examen physique, la patiente était apyrétique, tachycarde à 102 battements / minute avec une pâleur cutanéo-muqueuse et une pression artérielle à 120 / 80 mmHg. L'examen de la cavité buccale montrait des ulcérations au niveau du versant muqueux de la lèvre inférieure et une lésion ulcéro-bourgeonnante du bord latéral de la langue faisant 2 cm de grand axe (Figure 1). Les réflexes ostéotendineux étaient abolis aux membres inférieurs où on notait des ‘dèmes oedèmes blancs, mous et gardant le godet ainsi que quelques lésions purpuriques. Il n'y avait pas d'altération de la sensibilité superficielle ni profonde. L'examen de la marge anale montrait de multiples ulcérations péri-anales ainsi que des lésions purpuriques et nécrotiques (Figure 2). L'examen des urines à la bandelette montrait la présence de protéinurie et d'hématurie à deux croix. Le reste de l'examen physique était normal.

**Figure 3:**
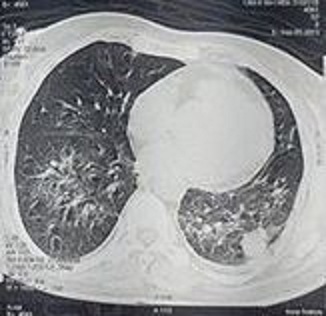
Coupe transversale d'une TDM thoracique montrant des plages étendues en verre dépoli avec une lésion nodulaire

A la biologie, il existait un syndrome inflammatoire biologique avec une VS à 70 la 1ère heure et une CRP à 110 mg /l. La numération formule sanguine montrait une hyperleucocytose à 15800 éléments / mm3 à prédominance de polynucléaires neutrophiles, sans hyperéosinophilie et une anémie normochrome normocytaire régénérative à 6.2 g/dl. On retrouvait également une insuffisance rénale rapidement progressive avec une créatinine passant de 373 à 443 micromol/l en 2 jours ainsi qu'une protéinurie à 1 g/24 heures. Le taux de prothrombine (TP) était à 45% sans cytolyse ni cholestase et avec un dosage normal du facteur V. La radiographie de thorax montrait de multiples opacités de condensation alvéolaire diffuses aux deux champs pulmonaires (Figure 3). Ces opacités, correspondaient au scanner thoracique, à des nodules intra-parenchymateux excavés associés à des plages en verre dépoli cadrant avec une hémorragie intra-alvéolaire et expliquant l'anémie retrouvée à la numération. Le reste de l'imagerie abdominale et pelvienne au niveau du scanner était normal. La rectoscopie montrait une muqueuse congestive siège d'ulcérations superficielles. Une biopsie de l'ulcération linguale était pratiquée montrant un remaniement inflammatoire non spécifique. La biopsie cutanée au niveau d'une lésion purpurique n’était pas également contributive, montrant des suffisions hémorragiques dermiques profondes entourées de capillaires à endothélium légèrement turgescent sans lésions de vasculite ni de leucocytoclasie. Une PBR n'a pu être programmée devant la gravité du tableau clinique et le TP bas. Les paresthésies et la faiblesse des membres inférieurs à l'effort étaient expliquées par une neuropathie périphérique détectée à l’éléctro-myogramme. Ainsi, devant l'hémorragie intra-alvéolaire à l'imagerie avec les nodules excavés, l'atteinte rénale, l'atteinte neurologique périphérique, l'hypothèse d'une vascularite systémique était très vraisemblable. La positivité des C-ANCA de spécificité anti-protéinase 3 confortait cette hypothèse et le diagnostic d'une granulomatose avec polyangéite était retenu. Le reste du bilan immunologique était par ailleurs négatif (AAN, cryoglobulinémie et sérologie des hépatites virales B et C).

Des bolus de méthylpredisolone, à raison de 1g/jour pendant 3 jours, étaient administrés et relayés par une corticothérapie orale associée à des bolus d'endoxan à la dose de 500 mg prescrits initialement de façon bi-mensuelle puis toute les 3 semaines. L’évolution était marquée initialement par une régression des lésions cutanées et muqueuses, une reprise d'un meilleur état général ainsi qu'une amélioration partielle de la fonction rénale (crétainine à 148 micromol/l à un mois de traitement), ainsi qu'une normalisation de la radio thorax, mais secondairement, à 2 mois d’évolution, la patiente est décédée à la suite d'un sepsis à point de départ urinaire compliqué d'un état de choc septique.

## Discussion

Nous rapportons l'observation d'une patiente de 57 ans, sans antécédents pathologiques notables, qui se présentait avec des rectorragies, des ulcérations péri-anales ainsi que buccales avec nécrose du bord latéral de la langue. L'anémie, l'aspect radiologique d'hémorragie intra-alvéolaire, l'insuffisance rénale rapidement progressive avec l'atteinte glomérulaire ainsi que la neuropathie périphérique ont fait penser à une vascularite systémique et notamment à une GPA, (maladie de Wegener). La positivité des C-ANCA venait renforcer cette hypothèse. Les manifestations cutanées sont fréquemment décrites au cours de cette vascularite et restent dominées par le purpura vasculaire nécrotique [4]. Ces manifestations peuvent cependant revêtir plusieurs aspects cliniques non spécifiques, s'intégrant le plus souvent à des formes actives de la maladie avec une atteinte multi-viscérale, mais elles sont rarement inaugurales [5]. Les aspects anatomopathologiques des lésions cutanées sont multiples et peuvent réaliser la triade caractéristique de la GPA: granulome tuberculoïde, nécrose et vascularite. Cette triade n'est cependant pas constante et la négativité des prélèvements biopsiques n’élimine pas le diagnostic [6]. L'atteinte ORL de la GPA est aussi fréquente et précoce mais c'est les atteintes nasales et sinusiennes qui prédominent [7]. Les manifestations buccales sont moins fréquentes et ne sont inaugurales que dans 2% des cas [8]. La gingivite hyperplasique représente l'atteinte la plus évocatrice de la GPA [9]. La nécrose de la langue n'est rapportée qu’à travers quelques rares cas cliniques [8–10]. Concernant l'atteinte digestive, elle s'observe chez 5 à 11% des patients [2, 11], mais le caractère révélateur est également rare [12]. Les ulcérations anales sont de même très peu décrites dans la littérature [3, 13, 14].

## Conclusion

La particularité de cette observation est représentée par le caractère inaugural de certaines manifestations inhabituelles de la GPA et notamment les ulcérations muqueueses endo-buccales et anales ainsi que les rectorragies. Une analyse clinique fine des signes associés est importante pour un diagnostic et une prise en charge adéquate.
